# Comparison of Support-Vector Machine and Sparse Representation Using a Modified Rule-Based Method for Automated Myocardial Ischemia Detection

**DOI:** 10.1155/2016/9460375

**Published:** 2016-01-26

**Authors:** Yi-Li Tseng, Keng-Sheng Lin, Fu-Shan Jaw

**Affiliations:** ^1^Department of Electrical Engineering, Fu Jen Catholic University, New Taipei City 24205, Taiwan; ^2^Institute of Biomedical Engineering, National Taiwan University, Taipei 10617, Taiwan; ^3^Graduate Institute of Communication Engineering, National Taiwan University, Taipei 10617, Taiwan

## Abstract

An automatic method is presented for detecting myocardial ischemia, which can be considered as the early symptom of acute coronary events. Myocardial ischemia commonly manifests as ST- and T-wave changes on ECG signals. The methods in this study are proposed to detect abnormal ECG beats using knowledge-based features and classification methods. A novel classification method, sparse representation-based classification (SRC), is involved to improve the performance of the existing algorithms. A comparison was made between two classification methods, SRC and support-vector machine (SVM), using rule-based vectors as input feature space. The two methods are proposed with quantitative evaluation to validate their performances. The results of SRC method encompassed with rule-based features demonstrate higher sensitivity than that of SVM. However, the specificity and precision are a trade-off. Moreover, SRC method is less dependent on the selection of rule-based features and can achieve high performance using fewer features. The overall performances of the two methods proposed in this study are better than the previous methods.

## 1. Introduction

Myocardial ischemia is the most common type of heart disease that is caused by a reduced blood supply to heart muscles. Cardiac tissues that lack oxygen begin to die, which results in myocardial infarction or heart attack and can lead to acute infarction and sudden death. To reduce mortality among patients who suffer from ischemic heart diseases, several important signs are used for early diagnosis. The most commonly used signs are those derived from electrocardiogram (ECG) signals, a noninvasive procedure [1–3].

During the last decade, long-term ECG recording and ambulatory ECG monitoring have been developed for monitoring cardiac diseases [4–6]. These techniques have improved the quality of ECG recordings and increased the possibility of detecting coronary syndromes in their early stages. The development of automated methods for detecting changes on ECGs has also become an important issue. When combined with monitoring techniques, these methods can indicate the early onset of acute myocardial ischemia and provide support for physicians in early diagnosis.

Ischemia often leads to ST segment and T wave changes on ECGs because it removes the delay between the repolarization of the endocardium and the epicardium. A hyperacute T wave will be observed first and will be followed by an ST elevation [2]. T wave inversions and other changes on ST segment will then occur [7]. Thus, automated detection methods have often been proposed to evaluate deviations in the ST-T complex, including ST segments and T waves.

Several methods have been proposed to detect ischemia on ECGs. These automated systems often comprise three parts: preprocessing, feature extraction, and classification [1]. Methods based on feature extraction, such as rule-based systems [8–12], principal component analysis (PCA) [13, 14], and Karhunen-Loeve transforms (KLT) [15, 16], were mostly derived based on medical knowledge. These methods often set a threshold for each selected feature by the experience of physicians and separate patients from normal subjects. However, these methods have low accuracy and are not adaptable to ECG signals obtained from different instruments or from different patients. In contrast, higher performance can be obtained using methods primarily based on classification, such as fuzzy logic systems [17, 18], artificial neural networks (ANN) [19, 20], genetic algorithms [21], support vector machines (SVM) [20, 22–25], multilayer perceptron (MLP) neural networks [26], and extreme learning machines (ELM) [27]. Although the methods based on classification often lack a medical knowledge basis and are used without rule-based features, they are more adaptable and have higher accuracies.

Previously published methods using rule-based techniques, artificial neural networks, and genetic algorithms all have good accuracies up to 90% [1]. Among the methods for classification, the SVM method has good performance with high accuracy and sensitivity [22–24]. Therefore, it has become one of the most popular methods for classifying abnormal ECG signals for ischemia detection. This method can classify data using an optimized hyperplane. It is a powerful machine-learning method for handling nonlinear data with small sample sizes.

Sparse representation-based classification (SRC) is a newly developed classification method. It has been successfully used for problems in pattern recognition, biometric, biomedical signal, and image processing [10, 24, 28–40]. In recent studies, it has been incorporated in the detection of several kinds of cardiac diseases such as premature ventricular contractions, arrhythmia, and ventricular ectopic beats with high detection rate close to 100% [37–40]. The SRC method assumed that signals could be sparsely represented by a linear combination of few basis elements, which is suitable to be utilized in blind source separation of biomedical signals. The SRC method is especially powerful for classifying data with large sample sizes. In contrast to the SVM method, the SRC method is less dependent on the features that are selected, which improves upon the drawbacks inherent in most classification methods. Using these two methods combined with a traditional rule-base method, our goal was to implement an automated method to detect myocardial ischemia.

In this paper, an automated method is established for the detection and analysis of myocardial ischemia and acute myocardial infarction. The analysis of ECG signals is implemented using SVM with rule-based vectors as the input feature space. A new SRC method is also included as a classification method to improve the performance of the existing algorithms. We describe these methods and provide an evaluation of their performance.

## 2. Methods

**Figure 1:**
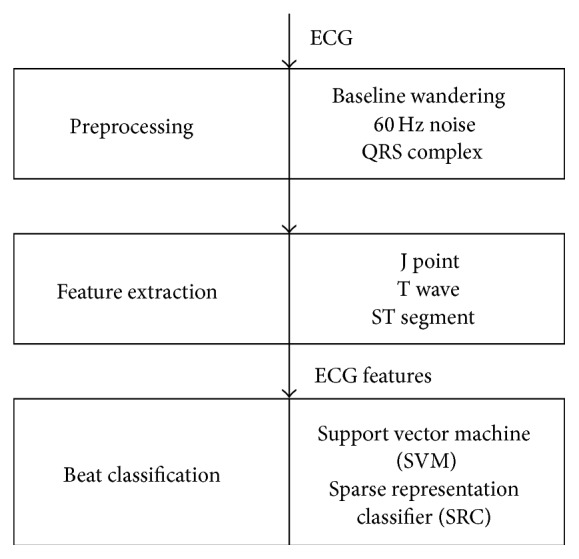
Block diagram of ECG processing for ischemia detection.

Two automated methods are developed for detecting cardiac ischemia using ECG signals. A schematic diagram of these methods is shown in Figure 1. The proposed methods are divided into three steps: (1) preprocessing, (2) feature extraction, and (3) beat classification. Details of these steps are described in the following subsections.

### 2.1. Preprocessing

**Figure 2:**
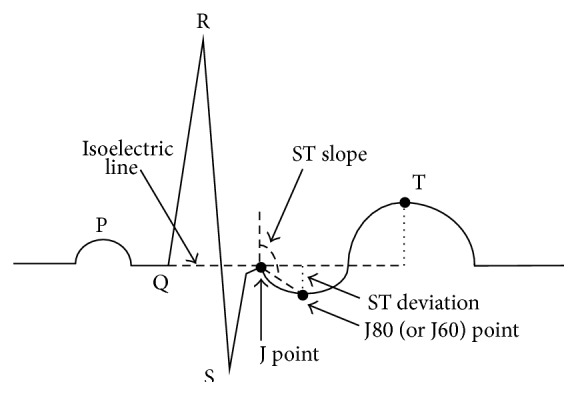
Features of each ECG beat.

The ECG signals are preprocessed by removing baseline wandering and 60 Hz noise. As shown in Figures 1 and 2 [21], QRS complexes are also detected in this step. These steps are implemented by the following procedures.

#### 2.1.1. Removal of Baseline Wandering and Smoothing

Recorded ECG signals are often accompanied by baseline wandering. This type of slowly varying noise can be eliminated by curve fitting. A cubic spline [23, 42, 43] is implemented using each isoelectric point of an ECG beat as a knot for optimal fitting of the wandering curve. An averaging filter is then introduced to smooth the ECG signal. This processing increases the accuracy of the following steps during feature extraction.

#### 2.1.2. Detection of the QRS Complex

The R peak of each beat shown in Figure 2 of an ECG signal is detected by the method developed by Benitez et al. [44, 45]. The first derivative of each ECG beat is calculated and then followed by a Hilbert transform and peak detection. This method can increase the ratio of the R peak to the T wave, which increases the detection rate of the QRS complex.

### 2.2. Feature Extraction

This stage is aimed at extracting the information in an ECG signal related to myocardial ischemia. Feature extraction is based on a rule-based method using essential information, including T amplitude and ST deviation. Based on this rule-based method [8] and clinical experience [7, 46], seven features are included in our method, as illustrated in Figure 2: ST deviation, ST slope, T amplitude, ST area, J80 amplitude, T magnitude, and T wave/R peak are computed and used as the features for detection.

To compute the T amplitude, ST deviation, ST slope, and other features, the basic components of an ECG beat, such as the J point and the isoelectric line shown in Figure 2 [9, 21], should be detected in advance. An edge-detection method first developed by Daskalov et al. in 1998 is used for recognizing these components [43]. This is suitable for obtaining a flat peak or a turning point by detecting a small interval whose slope is sufficiently small. The peaks of a T wave, P wave, J point as a turning point, and the isoelectric line are all detected using this method. The isoelectric line is determined from a 100 ms time interval before the R peak with a slope criterion, Cs < 2.5 *μ*V/ms within 20 ms, and thus the flat area in the ECG beat is found. The J point is calculated using the same method from a time interval after the R peak. Details of these procedures are provided in the paper by Daskalov et al. [43].

ST deviation is defined by subtracting the J80 (J + 80 ms) point from the isoelectric line. If the heart rate is >120 beats per minute, the J60 (J + 60 ms) point is used instead of J80. The ST slope is the steepness of the line formed by the J80 (J60) point and the J point. This feature indicates whether the ST segment is elevated or depressed.

The T amplitude is obtained by subtracting the peak of T wave from the isoelectric line. This indicates whether the T wave is normal or at risk of being hyperacute. A T inversion can also be detected.

After extracting these seven features, they are then fed into the SVM or SRC as the input vectors for beat classification.

### 2.3. Beat Classification

The extracted features are classified by SVM or SRC. These classification methods can divide the features into two groups: normal and abnormal with ischemia.

**Figure 3:**
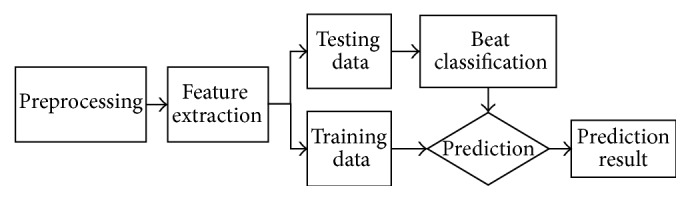
The training and testing datasets for classification.

To verify the classification results, the database used was first divided into training and testing data sets. As shown in Figure 3, the training set was used to train the SVM and SRC methods, and the testing set was used to validate the results after classification. The details of the two classification methods are described in the following subsections.

#### 2.3.1. SVM

SVM is a powerful machine learning method that can identify an optimized hyperplane with vectors in feature space divided by a maximal margin. One of the advantages of this classifier is that less training data needs to be used as compared to other methods. In addition, errors and complexity can be minimized [23, 47].

For linearly separable data, let *D* be an *n*-point training data set that is defined by

(1) $$\begin{array}{c} D={\left\{\;\left(\;x_{i},c_{i}\;\right)\;|\;x_{i}\in R^{p},c_{i}\in \left\{\;-1,1\;\right\}\;\right\}}_{i=1}^{n}, \end{array}$$

where *x*_*i*_ is the *i*th training vector that is classified in group *c*_*i*_. SVM attempts to obtain the maximum-margin hyperplane. This hyperplane can be expressed as

(2) $$\begin{array}{c} w\cdot x-b=0, \end{array}$$

where the vector *w* is a normal vector perpendicular to the hyperplane and *b*/‖*w*‖ is the offset of the hyperplane from the origin.

To derive the optimal hyperplane, the margin should be maximized by maximizing the distance 2/‖*w*‖, which minimizes ‖*w*‖. Because *w* · *x* − *b* ≥ 1 for the data in the first class and *w* · *x* − *b* ≤ −1 for data in the second class, the formula can be reduced to *c*_*i*_(*w* · *x* − *b* ≥ 1) ≥ 1 for all 1 ≤ *i* ≤ *n*. Therefore, optimization should be the solution of the original problem that minimizes ‖*w*‖ subject to *c*_*i*_(*w* · *x* − *b* ≥ 1) ≥ 1.

SVM can also deal with data that are not linearly separable using a radial basis function (RBF), which is used as the kernel function to create nonlinear classifiers. The details for nonlinear classification can be found in the paper by Burges [47]. These types of optimization problems can be resolved using the SVM toolbox function LibSVM [48] incorporated in MATLAB. The feature vectors described in the previous section can be classified into two classes using the SVM method. The optimal parameters C and G of RBF kernel are determined by grid search.

#### 2.3.2. Sparse Representation-Based Classification (SRC)

The basic idea of sparse representation is to represent the signal *y* ∈ *R* ^*n*^ using vectors from a dictionary *A* = {*a*_1_, *a*_2_, *a*_3_,…, *a*_*m*_}, where each *a*_*k*_ ∈ *R* ^*n*^ and *k* = 1,2, 3,…, *m*. For an overcomplete dictionary with *m* > *n*, the sparse representation attempts to attain a vector, *x*, in which *y* = *Ax* and ‖*x*‖_0_ is minimized. For ischemic and nonischemic beat classification, we used the features of the training data set extracted in Section 2.2 as the dictionary *A*. *y* can be represented by only a few vectors in the dictionary *A*, as shown by the following:

(3) x^0=arg min x0subject  to Ax=y,

where ‖·‖_0_, *l*_0_ norm denotes the number of nonzero entries. In this study, each testing data set of ECG beats is represented as signal *y* to solve (3). Although deriving a solution for (3) is NP-hard, it has been proved that a solution for (3) can be obtained by alternatively solving the *l*_1_ norm problem [49] as follows:

(4) x^1=arg min x1subject  to Ax=y,

where ‖*x*‖_1_ denotes the *l*_1_ norm. The solution for (4) approximates to that for (3), which means that ${\hat{x}}_{0}={\hat{x}}_{1}$ when the solution is sufficiently sparse. The formula for (4) is a convex optimization problem that can be solved by Basis Pursuit using linear programming. A MATLAB toolbox function, CVX [50], developed by Grant and Boyd is a powerful tool for solving this kind of problem.

### 2.4. Performance Evaluation

#### 2.4.1. European ST-T Database

The European Society of Cardiology ST-T (ESC ST-T) database [51] was used for training and testing the system. Cardiac beats from the ESC ST-T database were used to verify the method. There are 48 ECG signals from 2-hour recordings in the European ST-T database with ST and T wave deviations. A total of 462 cardiac beats were included. The dataset contained 231 normal beats and 231 ischemic beats, with 114 beats that included ST deviations and 117 with T wave abnormalities.

#### 2.4.2. Cross-Validation

The performance of the SVM and SRC methods was evaluated by cross-validation. A10-fold cross-validation was selected because this tends to provide a less biased estimation of accuracy [52]. Groups of training sets and testing sets were fed into the SVM and SRC classifiers, and accuracy was averaged after 10-fold cross-validation.

## 3. Results

The performance of the SVM and SRC methods was first evaluated separately using varying numbers of features. Then, the results from SVM and SRC were compared. Three indices were used to evaluate the performance: sensitivity, specificity, and precision. Sensitivity denotes the ability to detect ischemia, which is determined by the number of detected cases divided by all cases. Specificity is determined by the number of detected normal cases divided by all normal cases, which indicates the probability of correctly identifying “not ischemia.” Precision, also denoted as the positive predictivity, indicates the probability that a case classified as ischemia is in fact ischemia [53, 54]. Among these, sensitivity is the most widely used index for the evaluation of medical instrumentation. However, the other two indices are also important.

**Figure 4:**
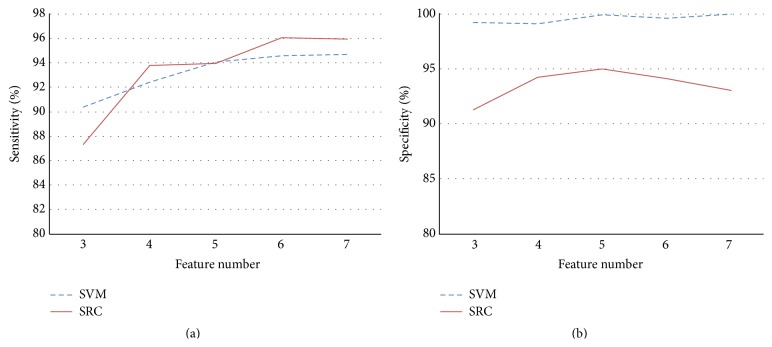
Sensitivity and specificity of the SVM and SRC methods using varying numbers of features.

Among the seven features used for training the system, we considered the best number of features to be used in the automated detection systems based on SVM and SRC methods. In addition to the three most important features, J80-J, ST slope, and T value, the four other features were included sequentially, after which the sensitivity and specificity were determined. As shown in Figure 4, the sensitivity was better when using six features for the SRC system, and the specificity was better when five features were included. By comparison to the SVM method, the SRC method achieved better results using fewer features.

The performance of the two methods was evaluated using 462 normal and abnormal beats from 48 cases. As shown in Table 1, the overall sensitivity and specificity were determined by averaging the results of 10-fold cross-validation using 90% of the data as the training set and 10% as the testing set. The sensitivity was only 94.81% when classified by SVM. For the SRC method, the sensitivity was up to 96.62%. However, the specificity for the SRC method was only 96.62% compared to 99.51% for SVM because there is a trade-off between sensitivity and specificity. The precision for SVM was slightly higher than for the SRC method.

## 4. Discussion

Two automated methods incorporating feature extraction and classification have been proposed for detecting abnormal ST segments and T waves in ECG beats. These abnormal ECG beats are early signs of myocardial ischemia. The feature extraction step is aimed at extracting the abnormal features of ECG beats, which is similar to the rule-based detection methods developed in previous investigations [8–10]. The feature extraction method is beneficial for obtaining abnormal features in consideration of medical advice, as in rule-based methods. However, the disadvantage of a rule-based method is that it is not an adaptive method. The rules defined in a rule-based method may be limited by a specific threshold that may not be appropriate for all data sets. By utilizing the classification methods after feature extraction, our method can overcome these limitations.

By combining the two classification methods, SVM and SRC, with a rule-based method, the proposed methods provide high sensitivity and accuracy. The sparse represented-based classification is a novel method that has provided good performance in the area of face recognition [55] and has recently been applied in the studies of biomedical signal processing [28, 30, 32, 33, 35, 38–40]. Mathews et al. have demonstrated a SRC method in 2015 to classify the abnormal ventricular ectopic beats (VEB) and supra VEB and reported high classification accuracy of 97.18% and 94.61%, respectively [37]. Baali and Mesbah have also proposed a method for arrhythmia classification based on SRC with the accuracy close to 100% [38]. For separating multiple types of abnormal heartbeats, Huang et al. have combined independent component analysis (ICA) with SRC to distinguish eight types of heartbeats and achieved the sensitivity range between 94.49 and 100% [39]. Furthermore, sparse method has been proved to be useful for enhancing the QRS complex and reducing the baseline wandering or muscle artefacts in ECG signals [40]. In this study, we are the first to incorporate the SRC method for ischemia heartbeat detection. We compared the results of ischemia beat classification using the SRC method with those of SVM, which was developed and commonly used during the last decade. The performances of SVM and SRC were both validated with quantitative estimations.

The results of the proposed rule-based feature extraction and SVM classification method were compared to those of the previous works. The SVM classification method has been widely used in the detection of myocardial infarction, arrhythmia, and physical activities recognition from ambulatory ECG signals [56, 57]. Although it is a blind classification method, diagnosis methods based on SVM often achieve high sensitivity and accuracy. The sensitivity of our SVM method was 94.81%, which was slightly higher than the SVM method proposed by Mohebbi and Moghadam in 2007 with a sensitivity of 92.31% [23]. Since the features extracted by the two methods are distinct, the results may also be varied. The features used in our method are fewer in number but are more closely related to medical indicators than the previous methods. The method proposed by Park et al. in 2012 has also shown high sensitivity of 94.1% [25]. Identical with the method proposed by Park et al., our method is designed with carefully specified features which are highly correlated with the clinical evidences observed in the ECG signals from patients with myocardial ischemia, such as ST elevation or the changes of T waves. In contrast, the method demonstrated by Mohebbi and Moghadam has utilized the whole ST segment as the training data for SVM without feature selection. This may be a reason why our proposed method has shown higher sensitivity. The step of rule-based feature extraction is therefore essential and important in the improvement of the accuracy for the diagnostic methods.

In addition to the SVM method, other previous methods have proved to provide good performance for classifying ischemia ECG beats, such as fuzzy expert systems and genetic algorithms. In Table 2, the previous studies using the same testing database and having the sensitivity reported are compared with our study. The European ST-T database is used in all of these methods. Most of the traditional rule-based methods (the first two methods shown in Table 2) have demonstrated lower accuracy. Hybrid detection methods combining the rule-based criteria and classifiers, such as the multicriteria decision analysis proposed by Goletsis et al. in 2004 [21], provide comparable sensitivity to the blind classifications ones. A fuzzy expert system is also used for ischemia beats classification by Exarchos et al. in 2007. The sensitivity of this fuzzy expert system was about 91% [18], which was slightly lower than the SVM and SRC methods. The blind classification methods based on SVM or other algorithms often demonstrate higher sensitivity and accuracy as shown in Table 2. However, hybrid methods with rule-based feature extracted are able to provide both high accuracy and clinical and theoretical support. According to Table 2, the hybrid methods with rule-based feature selection and classification based on SVM and SRC proposed in this study still exhibited higher sensitivities.

The proposed methods can detect ischemia with high sensitivity. As shown by our results, the sensitivity was higher for the SRC-based method compared to SVM. However, the specificity and precision of SVM were better than that of SRC because there is a trade-off between sensitivity and specificity. The number of features used is another consideration when comparing these two methods. As shown in Figure 4, the sensitivity for SVM was better when more features were included. In contrast, there was an optimal number of features for the proposed SRC method, which was six. This result shows that SRC is actually less dependent on the number of features. Furthermore, the computational complexity could be reduced because fewer features were required in the SRC method. Unlike SRC, the performance of SVM improved when using more features as its input vectors, which may be a waste of computational time. In summary, the overall performances of the proposed methods are higher than those of the previous methods.

## 5. Conclusion

In this study we presented two automated methods for detecting myocardial ischemia. These two methods are implemented using a modified knowledge-based method, including rule-based feature extraction and novel classification methods. We compared the performances of these two classification methods, SVM and SRC, using knowledge-based features as the input vectors. The SRC method exhibited higher sensitivity than the SVM method using distinct numbers of rule-based features. Although the specificity and precision are a trade-off when compared with the SVM method, which is a well-known method in the area of ischemia detection, SRC could still achieve a higher sensitivity. Furthermore, fewer features are required for SRC. In conclusion, the SRC method is a comparable classification method with high sensitivity that is less dependent on rule-based features and could be used in the detection of biomedical signals. To our knowledge, the proposed method is the first study with SRC method incorporated to detect myocardial ischemia. Quantitative evaluation using different number of rule-based features and classification methods is provided to validate the advantage of the proposed hybrid method and the superior performance of the new SRC method. The results suggest the benefit of using the SRC method as a diagnostic tool in myocardial ischemia detection. Furthermore, the consistent findings with the previous studies suggest that a hybrid detection method with rule-based feature selection is essential in future diagnostic applications.

## Figures and Tables

**Table 1:** Comparison of the support vector machine (SVM) and sparse representation-based classification (SRC) methods.

	Sensitivity	Specificity	Precision
Support vector machine (SVM)	94.81%	99.51%	99.9%

Sparse representation-based classification (SRC)	96.62%	96.62%	99.49%

**Table 2:** Comparison of the classification results from previous studies for ischemic beat detection.

Method	Sensitivity (%)
RMS difference series [12]	85
Rule-mining based [11]	87
Back propagation network [41]	89
Artificial neural networks (ANN) [19]	90
Principal components analysis and neural networks [13]	90
Genetic algorithm and multicriteria [21]	91
Fuzzy expert system [18]	91
SVM [23]	92
Rule-based [9]	92
Knowledge-based [8]	94
Kernel density estimation (KDE) [25]	94
SVM [25]	94
SVM (this work)	94.81%
Sparse representation-based classification (SRC) (this work)	96.62%
